# *Cinnamomum zeylanicum* (Ceylon cinnamon) as a potential pharmaceutical agent for type-2 diabetes mellitus: study protocol for a randomized controlled trial

**DOI:** 10.1186/s13063-017-2192-0

**Published:** 2017-09-29

**Authors:** Priyanga Ranasinghe, Priyadarshani Galappaththy, Godwin Roger Constantine, Ranil Jayawardena, Hasitha Dhananjaya Weeratunga, Sirimal Premakumara, Prasad Katulanda

**Affiliations:** 1 Department of Pharmacology, Faculty of Medicine, University of Colombo, Colombo, Sri Lanka; 2 Department of Clinical Medicine, Faculty of Medicine, University of Colombo, Colombo, Sri Lanka; 3 Department of Physiology, Faculty of Medicine, University of Colombo, Colombo, Sri Lanka; 4 Institute of Health and Biomedical Innovation, Queensland University of Technology, Brisbane, QLD Australia; 5 Industrial Technology Institute, Colombo, Sri Lanka

**Keywords:** *Cinnamomum zeylanicum*, Ceylon cinnamon, Diabetes, Sri Lanka, Adults

## Abstract

**Background:**

Previous studies have explored the anti-diabetic effects of *Cinnamomum cassia* extract in vivo and in vitro. However, there are no studies at present exploring the effects of the indigenous species of Sri Lankan cinnamon (*Cinnamomum zeylanicum*) in patients with diabetes mellitus. The present study aims to evaluate the potential effects of *Cinnamomum zeylanicum* extract as a pharmaceutical agent in patients with type-2 diabetes mellitus.

**Methods/design:**

The study will be conducted as a randomized, double-blind, placebo-controlled clinical trial for a period of 4 months at the Medical Clinic, University Medical Unit, National Hospital of Sri Lanka. A total of 210 subjects with diabetes, in three equal groups, will be recruited for the study. The patients will be randomized in a 1:1:1 ratio according to the method of block randomization and the subjects will be randomly and equally assigned into two test groups (*n* = 70 each) and one placebo group (*n* = 70). The population will be stratified at randomization based on age, gender and disease severity. The treatment drug is a capsule containing *Cinnamomum zeylanicum* extract as the active ingredient and the placebo capsule will contain lactose monohydrate. Two doses of *Cinnamomum zeylanicum* extracts (250 mg and 500 mg of the cinnamon extract) will be used. The study drugs will be double blinded to both investigators and participants. The visits and the evaluations will be done as follows: screening (visit 0), 1 month (visit 1), 2 months (visit 2), 3 months (visit 3) and 4 months (visit 4). The following primary outcome measures will be evaluated: glycosylated hemoglobin (HbA_1_c), fasting plasma glucose (FPG) and serum insulin. Secondary outcome measures include: Body Mass Index (BMI) and other anthropometric parameters, blood pressure, total cholesterol, low-density lipoprotein cholesterol (LDL), high-density lipoprotein cholesterol (HDL) and triglycerides (TAG). Data will be analyzed using SPSS version 14.

**Discussion:**

We describe the protocol for a clinical trial design evaluating the effects of *Cinnamomum zeylanicum* (Ceylon cinnamon) in patients with type-2 diabetes mellitus. The result of the present study, positive or negative, should provide a step change in the evidence guiding current and future policies regarding the use of cinnamon dietary supplementation in patients with diabetes.

**Trial registration:**

Sri Lanka Clinical Trials Registry (SLCTR), identifier: SLCTR/2017/010 (http://slctr.lk/trials/714). Registered on 5 April 2017; study protocol version 3.1 21 March 2017.

**Electronic supplementary material:**

The online version of this article (doi:10.1186/s13063-017-2192-0) contains supplementary material, which is available to authorized users.

## Background

Diabetes mellitus is a leading cause of morbidity and mortality worldwide, with an estimated 80% of the world population with diabetes living in lower- and middle-income countries (LMICs) like Sri Lanka [1, 2]. Most patients with the disease have type-2 diabetes, characterized by insulin resistance and β-cell dysfunction, leading to ultimate pancreatic β-cell failure [3]. South Asians are known to have an increased predisposition for type-2 diabetes, due to genetic, biological and lifestyle factors [4, 5]. The causes of type-2 diabetes are multifactorial and includes both genetic and environmental factors [6]. Diet plays an important role on the incidence, severity and management of type-2 diabetes [7]. This has resulted in numerous studies focusing on dietary components that are beneficial either in the prevention and/or treatment of type-2 diabetes and the findings of these individual studies have been summarized in systematic reviews [8–10].

Cinnamon is one such dietary component that has been shown to contain biologically active substances that regulate blood glucose by insulin-mimetic properties, which enhances glucose uptake by activating insulin receptor kinase activity, auto-phosphorylation of the insulin receptor and glycogen synthase activity [11]. Cinnamon has two main varieties, *Cinnamomum cassia* (also known as *Cinnamomum aromaticum*) and *Cinnamomum zeylanicum*. Sri Lanka produces the largest quantity and best quality of cinnamon. *Cinnamomum zeylanicum*, also known as Ceylon cinnamon (the source of its Latin name, zeylanicum) or “true cinnamon” is indigenous to Sri Lanka [11]. One important difference between true cinnamon and cassia cinnamon is their coumarin content [12]. Coumarins posses strong anticoagulant properties and can have potentially toxic effects on the liver [13]. The coumarin content in Ceylon cinnamon is negligible and is not known to cause detrimental health effects, whereas the coumarin level in *Cinnamomum cassia* is much higher and can cause health risks if consumed in larger quantities on a regular basis [12]. Several countries have restricted the regular usage of *Cinnamomum cassia* as a result of this potential health hazard associated with high levels of coumarins [12].

However, it is important to note that previous studies have explored the anti-diabetic effects of *Cinnamomum cassia* extract in vivo and in vitro [14–18]. At present there are no studies exploring the effects of the indigenous species of Sri Lankan cinnamon (*Cinnamomum zeylanicum*) on diabetes mellitus in vivo in humans. Since, *Cinnamomum zeylanicum* has been shown to contain negligible amounts of coumarin, it may be possible that Ceylon cinnamon could be used in higher doses without toxic effects for longer durations [12, 19]. A phase-1 clinical trial conducted by our study team, where *Cinnamomum zeylanicum* was given as a regular daily supplementation to healthy adults for a period of 3 months, failed to reveal any significant adverse effects (unpublished data). The present paper describes the study protocol for a randomized, double-blind, placebo-controlled clinical trial evaluating the efficacy of *Cinnamomum zeylanicum* (Ceylon cinnamon) in patients with type-2 diabetes mellitus.

## Methods/design

**Fig. 1:**
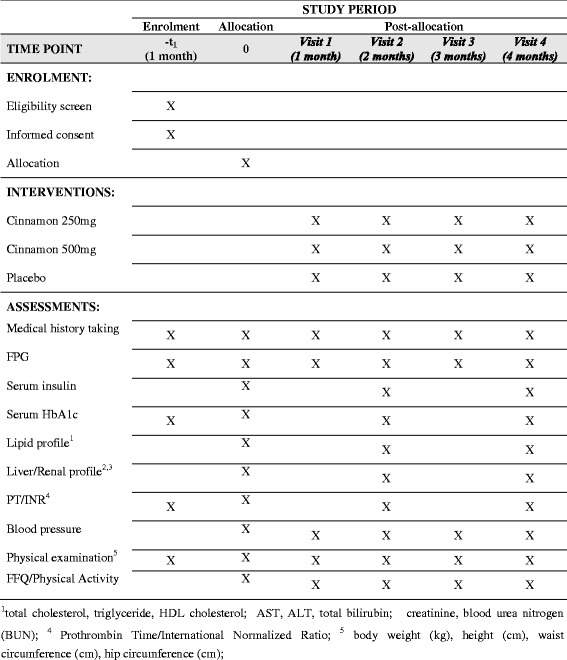
Summarized study schedule at each visit in the clinical trial

This protocol was written following the Standard Protocol Items: Recommendations for Interventional Trials (SPIRIT) Checklist (see Additional file 1) [20]. The schedule of trial enrollment, interventions and assessments is presented in Fig. 1.

### Objectives and hypothesis

Hypothesis: we hypothesize that the glycemic control of people with diabetes mellitus who are treated with *Cinnamomum zeylanicum* (Ceylon cinnamon) in addition to conventional treatment with oral hypoglycemic agents will be better than that of the control group treated only with oral hypoglycemic agents. We also hypothesize that other diabetes-related metabolic parameters (total cholesterol, triglycerides (TAG), high-density lipoprotein (HDL) cholesterol, low-density lipoprotein (LDL) cholesterol, and blood pressure) will be improved in the treatment group in comparison to the control group.

Objectives: the study evaluates the potential effects of *Cinnamomum zeylanicum* extract as a pharmaceutical agent in patients with type-2 diabetes in Sri Lanka. In addition to that the study evaluates the effects on serum lipids, anthropometric parameters, blood pressure and appetite and also studies the potential effects of regular administration of *Cinnamomum zeylanicum* extracts on liver and kidney function and the occurrence of self-reported side effects in patients with diabetes.

### Study design and setting

The study will be a phase-II/III, randomized, double-blind, placebo-controlled clinical trial. It will be conducted at the Medical Clinic, University Medical Unit, National Hospital of Sri Lanka for a period of 4 months, assessing the effects of the daily supplementation of capsules containing *Cinnamomum zeylanicum* in patients with type-2 diabetes mellitus. The Medical Clinic, University Medical Unit, National Hospital of Sri Lanka has around 400–500 patients with diabetes, attending the clinic on a monthly basis. The clinic operates 2 days per week and during all 4 weeks of a month. The duration of the trial will be 4 months, based on the evidence from previous research conducted by the investigators, and this duration is expected to be sufficient to identify changes in the primary glycemic outcomes (glycosylated hemoglobin (HbA1c), fasting plasma glucose (FPG) and serum insulin) assessed during the clinical trial [21].

### Sample size

The sample size was calculated using one-way analysis of variance (ANOVA), by the formula given below;

$$ {n}_A=\left({\sigma^2}_A+{\sigma^2}_B/\kappa \right){\left(\frac{z_1{{{{}_{-}}_{\alpha}}_{/}}_{\tau }+{{z_1}_{-}}_{\beta }}{\mu_A-{\mu}_B}\right)}^2 $$

- *n*_*A*_ – sample size in one group
- *σ*_*A*_ – standard deviation of group A
- *σ*_*B*_ – standard deviation of group B
- *κ* – sampling ratio
- *α* – type-I error
- *τ* – number of pairwise comparisons
- *β* – type-II error
- *μ*_*A*_ – mean of group A
- *μ*_*B*_ – mean of group B

The number of patients required in a group for determination of a 0.75% reduction of HbA_1_c in a treatment group (*μ*_*A*_ *–* 7.5%), in comparison with the placebo group (*μ*_*B*_ *–* 6.75%) at 80% power (*β*), 95% confidence level (*α* – 0.05), for three pairwise comparisons (*τ*), with a sampling ratio of 1 (*κ*) is 62 in one group according to the above formula. The standard deviation of the HbA_1_c (*σ*_*A*_ and *σ*_*B*_) was taken as 1.4 based on evidence from previous research among Sri Lankan patients with diabetes [22]. Hence, the total sample required for the three groups will be 186. Therefore, a total of 210 subjects with diabetes in three equal groups will be recruited for the study, allowing for a dropout rate of 10%. Subjects will be randomly and equally assigned into two test groups (*n* = 70 each) and will receive either a *Cinnamomum zeylanicum* oral capsule (250 mg or 500 mg) or an identical placebo daily for 4 months.

### Population

Diabetes is defined as the presence of a FPG ≥ 126 mg/dl or a plasma glucose level 2 h post oral glucose load of ≥ 200 mg/dl, or both. Participants will be recruited on a voluntary basis from a cohort of patients with diabetes mellitus attending the Medical Clinic, University Medical Unit, National Hospital of Sri Lanka. Informed written consent will be obtained from all study participants. Details of the inclusion and exclusion criteria are given below.

### Inclusion and exclusion criteria

#### Inclusion criteria

- Age 18–70 years
- Diagnosed type-2 diabetes mellitus during the last 5 years
- Currently only taking metformin or a sulphonylurea, or both (less than or equal to two oral hypoglycemic agents) for the last 3 months
- HbA_1_c 6.5–8.0% and or FBS 126–200 mg/dl

#### Exclusion criteria

- Patients with an allergy to cinnamon
- Alcohol consumption > 20 g/day
- Patients with diagnosed alcoholic liver disease (ALD), cirrhosis or abnormal baseline liver function tests
- Patients using insulin therapy
- Patients with raised baseline serum creatinine level (>1.5 mg/dl in men or > 1.2 mg/dl in women)
- Lactation, pregnancy or unwillingness to use an effective form of birth control for women of childbearing years
- Patients with symptoms suggestive of peptic ulcer disease and those with a past history of peptic ulceration
- Patients with any malignancy
- Patients with unrelated chronic illness
- Patients with cardiac, liver or respiratory failure
- Patients with bleeding disorders and who are taking coumarin derivatives (e.g., warfarin)
- Any condition that, in the opinion of the primary investigator, would contraindicate the patient’s participation

#### Suspension criteria

- Subject’s demand to discontinue the study
- Serious adverse events or unusual changes in clinical test results
- Principal investigator’s decision to terminate the study (low rates of compliance, complications or inability to tolerate the study for various reasons)

### Randomization

Randomization for the parallel treatment arms will be carried out by the principal investigator after checking the inclusion and exclusion criteria. The patients will be randomized in a 1:1:1 ratio according to the method of block randomization with a block size of 15. The population will be stratified at randomization based on, age (below 50 years and 50 years and over), gender and the severity of the disease (HbA_1_c 6.5–7.5% and 7.5–8.0%) to ensure equal distribution of these variables in the three arms. The randomization sequence would be generated using the SPSS statistical software package (version 14.0).

### Blinding

The investigators and patients are blind to the treatment allocations. The medication will be delivered in similar packets and labels, each with its own sequence number. The allocation sequence number will be generated by one of the investigators not involved in managing patients. Envelopes containing a monthly supply of either placebo or cinnamon will be prepared according to the randomization sequence and supplied to the patients by the principal investigator or research assistants when they are randomized to the trial.

### Interventions

The treatment drug is a capsule containing *Cinnamomum zeylanicum* extract as the active ingredient; it will have a white-colored body and a capsules. Two doses of cinnamon will be used in the present study (250 mg and 500 mg) based on data arising from previous research [17, 21]. Stem bark of *Cinnamomum zeylanicum* will be extracted to distilled water using Soxlet apparatus and the resulting hot-water extract will be freeze-dried to obtain a crude water extract from which the capsules will be prepared. The placebo capsule will contain lactose monohydrate and stearic acid. The weight of each cinnamon oral capsule will be 300 mg (cinnamon + inert filler material). The placebo oral capsule will be identical in shape, size, weight and texture to the *Cinnamomum zeylanicum* oral capsule. In order to mask the aroma, cinnamon quills would be put into packets containing both the placebo and cinnamon capsules. Each group would be given two oral capsules before breakfast for daily usage for a period of 4 months (Table 1). Capsules will initially be given at visit 0 for a period of 1 month, and subsequently a monthly supply will be given to participants at each clinic visit.

**Table 1:** Study groups in the clinical trial

Group	Number of capsules (1 = 250 mg of cinnamon)		
	Cinnamon	Placebo	Total
Group 1 (250 mg of cinnamon)	1	1	2
Group 2 (500 mg of cinnamon)	2	0	2
Group 3 (placebo)	0	2	2

### Study groups

#### Treatment groups

To evaluate a dose-response relationship, the phase-II study will include two treatment groups. The groups will receive *Cinnamomum zeylanicum* doses of either 250 mg or 500 mg based on results arising from previous research:

1. Group 1 – *Cinnamomum zeylanicum* dose 250 mg daily
2. Group 2 – *Cinnamomum zeylanicum* dose 500 mg daily

#### Control group

3. Group 3 – placebo group

### Study period

The study will be for a period of 4 months and the visits and the evaluations will be done as follows: screening (visit 0), 1 month (visit 1), 2 months (visit 2), 3 months (visit 3) and 4 months (visit 4) (Fig. 1).

### Outcomes

#### Primary outcome index

Primary glycemic outcomes assessed would be the HbA_1_c, FPG and serum insulin.

#### Secondary outcomes

1. Insulin resistance would be measured by the Homeostasis Model of Assessment-Insulin Resistance (HOMA-IR) calculations based on fasting blood glucose and fasting serum insulin levels. Evaluation of β-cell function would occur in vivo (HOMA-B calculation) and in vitro
2. Anthropometric assessment such as body weight, height, Body Mass Index (BMI), waist circumference (WC) and hip circumference (HC)
3. Measurement of systolic blood pressure (SBP) and diastolic blood pressure (DBP)
4. Measurement of lipid profile (total cholesterol, LDL cholesterol, HDL cholesterol and TAG)

#### Safety Assessment Index

The following information will be recorded/measured for the safety assessment: vital signs, general medical examination, kidney function tests, liver function tests (aspartate aminotransferase (AST), alanine aminotransferase (ALT), prothrombin time/International Normalized Ratio (PT/INR)) and adverse events. Liver profile and kidney profile will be done at the screening visit (visit 0), 2 months (visit 2) and at the completion of the study (visit 4).

### Procedures

#### Recruitment

Patients will be recruited on a voluntary basis from a cohort of patients with diabetes mellitus who are attending the Medical Clinic, University Medical Unit at the National Hospital of Sri Lanka. Those who consent to participate in the trial will visit the trial site voluntarily.

#### Study schedule

This involves measuring HbA_1_c, FPG and serum insulin according to the schedule described in Fig. 1. In addition, serum lipid profile (total cholesterol, triglycerides, LDL cholesterol and HDL cholesterol), liver profile (AST, ALT and PT/INR) and kidney profile (blood urea and serum creatinine) would be measured at baseline, 2 months and on completion of the study. The details of items which will be measured at every visit are described in Fig. 1.

### Measurement tools

#### Anthropometric measurements

Height will be measured using Harpenden pocket stadiometers (Chasmors Ltd., London, UK) to the nearest 0.1 cm. Body weight will be measured in light, indoor clothing to the nearest 0.1 kg using a SALTER 920 digital weighing scale (Salter Ltd., Tonbridge, UK). Waist circumference will be measured at midway between the iliac crest and the lower costal margin at the end of normal expiration using a flexible, plastic tape to the nearest 0.1 cm. Similarly, the HC also will be measured at widest part of the buttocks at the intertrochanteric level to the nearest 0.1 cm. All anthropometric measurements will be made by trained personnel.

#### Dietary measurements

A culturally validated Food Frequency Questionnaire (FFQ) will be used to obtain habitual intake of calories, macronutrients and micronutrients [23].

#### Physical activity

Physical activity will be evaluated by using the International Physical Activity Questionnaire (IPAQ) short version [24].

#### Clinical examination

Seated blood pressure is to be recorded on two occasions after at least a 10-min rest using an Accoson mercury sphygmomanometer (Accoson Healthcare, Asia-Pacific Region, Singapore).

#### Other

In addition to the above data, sociodemographic data, drugs and data on side effects will also be gathered via a questionnaire.

### Compliance calculation

Subjects are asked to return any remaining drugs and their compliance will be evaluated by using the formula given below:

$$ \mathrm{Compliance}\left(\%\right)=\left[\left(\mathrm{distributed}\kern0.17em \mathrm{drugs}-\mathrm{remaining}\kern0.17em \mathrm{drugs}\right)/\mathrm{distributed}\kern0.17em \mathrm{drugs}\times 100\right] $$

### Statistical analysis

Parametric (e.g., ANOVA) and non-parametric (e.g., Kruskal-Wallis test) statistical tests will be applied using the SPSS version 14 (SPSS Inc., Chicago, IL, USA) and Stata/SE 10.0 (Stata Corporation, College Station, TX, USA) for the data analysis. Data will be entered by a minimum number of dedicated staff and saved in a dedicated computer with password protection. For each of the outcomes, multilevel regression analysis will be used to examine differences between trial arms. For binary outcomes the model will be logistic and, for continuous outcomes, the model will be linear regression. All analyses will follow intention-to-treat principles and a prespecified analysis plan. Where appropriate, sensitivity analyses will be conducted (for example, control for additional covariates; and bootstrapped *p* values for skewed outcomes). In the case of missing data values, we will apply mean imputation and regression imputation where rates are low, and consider multiple imputations where they exceed 10%. Since multiple tests are performed simultaneously, a Bonferroni correction will be made during analysis to determine the critical *P* value for significance.

### Adverse effect evaluation

Studies have shown that the median lethal dose value (LD_50_) of orally administered cinnamon in animals is 1850 ± 37 mg/kg [25]. Hence, this is equivalent to a human dose of 11.4 ± 0.2 g/kg. Previous studies on *Cinnamomum cassia* have not documented any probable adverse effects related to the regular use of cinnamon in humans, in spite of *Cinnamomum cassia* containing potentially hepatotoxic coumarin levels [17, 26–28]. The present study would be conducted using *Cinnamomum zeylanicum* which is known to contain only minimal coumarin levels [12]. Furthermore, a recent meta-analysis of preclinical trials on Ceylon cinnamon has not shown any significant adverse effects in animals [11]. The type of adverse events expected are likely to be minor in nature, and possibly only related to gastric irritation. Hence, patients with symptoms suggestive of peptic ulcer disease, and those with a past history of peptic ulceration, will be excluded. Hypoglycemia is also possible as a pharmacodynamic interaction between cinnamon and oral hypoglycemic drugs. However, in the event of a probable adverse reaction the following precautions would ensure timely identification and management of patients:

1. Reporting – mechanisms would be set up to ensure direct reporting of probable adverse events to investigators by patients (via telephone available 24 h a day on all days)
2. During follow-up visits probable adverse events would be noted by history and examination and investigated in detail. All adverse effects observed will be documented in the Case Record Form (CRF)
3. Any serious adverse event as defined in Good Clinical Practice (GCP) guidelines will be reported to the National ADR Monitoring Center at the Department of Pharmacology, Faculty of Medicine, University of Colombo within 24 h, the Ethics Committee, Faculty of Medicine, University of Colombo and the Clinical Trials Subcommittee of the Drug Regulatory Authority within 1 week
4. A Data Safety Monitoring Board (DSMB) will evaluate all the adverse events at regular intervals. The DSMB will comprise three members, inclusive of a clinical pharmacologist, a statistician and a physician. The DSMB will be independent of the trial investigators
5. Investigations – liver and kidney functions would be assessed as detailed above
6. Management – in the events of an adverse reaction requiring in-hospital management, the facilities and expert management would be available at the University Medical Unit, National Hospital of Sri Lanka
7. Termination of study – the complete clinical trial will be terminated prematurely if there is evidence that the safety of the trial participants can no longer be assured or new scientific information arises during the course of the clinical trial regarding patient safety

### Data collection

After filling out the CRF, data collection will be performed according to the standard operating procedures (SOPs) by the trained clinical research associates (CRAs).

### Data management and monitoring

Storage: data will be entered by a minimum number of dedicated staff and saved in a dedicated computer with password protection. Samples will be stored in a secure facility, with measures taken to ensure that specimens are kept under correct and constant conditions at all times when in storage. Expert staff that has been trained specifically in sample storage and transportation would ensure that all regulatory issues are properly handled. Storage technologies with the capability of monitoring the temperature of samples around the clock would be utilized.

Sample disposal: after each analysis is completed and with the approval of the principal investigator, the samples stored in the storage facility may be disposed of by the sample custodian. A Sample Disposal Sheet is completed and kept for further reference.

### Dissemination of study finding

The results of the above study will be published in local and international, peer-reviewed journals and presented at international conferences and clinical meetings.

### Ethical considerations

The study has been approved by the Ethics Review Committee of the Faculty of Medicine, University of Colombo, Sri Lanka. The trial is also registered at the Sri Lanka Clinical Trials Registry (SLCTR/2017/002). The study will be conducted in compliance with the Declaration of Helsinki and the GCP guidelines. Any change in trial protocol will be notified to the relevant regulatory authorities and trial participants, with re-consent being taken from participants, if required.

## Discussion

In this article we describe the protocol for a clinical trial design evaluating the effects of *Cinnamomum zeylanicum* (Ceylon cinnamon) in patients with type-II diabetes mellitus. To our knowledge this is one of the first randomized controlled trials evaluating the effects of supplementation of Ceylon cinnamon in patients with diabetes. Hence, the present study will provide the required foundation for future, large-scale, multicentered clinical trials. Given the present interest in using a variety of nutritional supplements to enhance glycemic control in patients with diabetes, properly planned and methodical scientific studies are a timely necessity. However, in relation to cinnamon, presently there are no well-designed randomized control trials conducted for a satisfactory time period to support/refute this argument. Hence, the result of the present study, positive or negative, should provide a step change in the evidence guiding the current and future policies regarding the use of cinnamon dietary supplementation in patients with diabetes.

### Trial status

Enrollment for the trial has not yet started (scheduled date – 1 December 2017).

## Additional file

Additional file 1: SPIRIT 2013 Checklist: recommended items to address in a clinical trial protocol and related documents. (DOC 121 kb)

## Abbreviations

ALD: Alcoholic liver disease
ALT: Alanine aminotransferase
AST: Aspartate aminotransferase
CRA: Clinical research associate
CRF: Case Report Form
FPG: Fasting plasma glucose
HDL: High-density lipoprotein
HOMA-IR: Homeostasis Model of Assessment-Insulin Resistance
IPAQ: International Physical Activity Questionnaire
LDL: Low-density lipoprotein
SOP: Standard operating procedure
SPSS: Statistical Package for the Social Sciences
